# Ischaemic heart disease and Cancer: competing malignant conditions

**DOI:** 10.1186/s12872-020-01539-5

**Published:** 2020-05-27

**Authors:** Alexandra C. Murphy, Anoop N. Koshy, Matias B. Yudi

**Affiliations:** 1 Department of Cardiology, Austin Health, Austin Hospital, 145 Studley Road, Heidelberg, Melbourne, Victoria 3084 Australia; 2 Department of Medicine, The University of Melbourne, Melbourne, Victoria Australia

## Abstract

**Background:**

The growing population of cancer survivors and their high frequency of cardiovascular disease have resulted in a dramatic increase in cancer patients requiring cardiovascular intervention. However, there is a lack of evidence to guide optimal management in this complex population who have historically been excluded from cardiovascular trials.

**Discussion:**

We review the recently published meta-analysis by Roule et al. The findings of this analysis demonstrate that patients with cancer presenting with acute coronary syndrome (ACS) have increased rates of in-hospital cardiovascular mortality, bleeding and one-year cardiovascular mortality. All-cause mortality measured in-hospital and at one-year were also significantly greater in cancer patients as was all-cause mortality in cancer patients that undergo percutaneous coronary intervention (PCI). In contrast to the short-term outcomes, rates of long-term cardiovascular mortality did not differ significantly between groups.

**Summary:**

Patient-specific assessment of risk, accounting for disease characteristics and treatment, and close communication with oncology providers is vital in defining optimal treatment strategies in this population.

## Background

Due to advancements in modern cancer therapy we are now seeing higher rates of cure and the conversion of a terminal illness into a chronic disease [1]. The growing population of cancer survivors and their high frequency of cardiovascular disease have resulted in a dramatic increase in cancer patients requiring cardiovascular intervention [2]. The causal relationship between acute coronary syndrome (ACS) and cancer can be partially attributed to the shared risk factor profile between the two disease states, such as obesity, diabetes and smoking [3]. Additionally, the pro-inflammatory and hypercoagulable state associated with many cancers and the acceleration of atherosclerosis caused by certain cancer therapeutics may also contribute to the observed increased prevalence of ACS [4–6]. Optimisation of cardiac risk factors in this population is of the upmost importance, both for the prevention of cardiovascular events and the future development of cancer therapy related cardiotoxicity and is a hallmark of modern Cardio-Oncology care. Patients with cancer presenting with ACS pose a diagnostic and therapeutic challenge. Although initial assessment should mirror that of the general population, there is a lack of evidence to guide optimal management in this complex population who have historically been excluded from cardiovascular trials [7–9].

## MAINT text

In their systematic review and meta-analysis, Roule et al. assessed the influence of cancer on early and late all-cause and cardiac mortality in the setting of ACS including ST-elevation myocardial infarction (STEMI) and/or percutaneous coronary intervention (PCI) [10]. This is an important contribution to the field, where there is limited evidence to guide practice. The findings of this analysis demonstrate that patients with cancer presenting with ACS have increased rates of in-hospital cardiac mortality, bleeding and one-year cardiac mortality. All-cause mortality measured in-hospital and at one-year were also significantly greater in cancer patients as was all-cause mortality in cancer patients that undergo PCI. In contrast to the short-term outcomes, rates of long-term cardiac mortality did not differ significantly between groups.

The results of this study highlight three important points. Firstly, of the patients examined, those with cancer were generally older, with a greater burden of co-morbidities which has important implications for ACS prognosis irrespective of malignancy status.

Secondly, cancer patients have higher rates of cardiac mortality, measured in-hospital and at one-year, than non-cancer patients. Of interest, cancer patients who underwent PCI had comparable cardiovascular mortality to non-cancer patients. Although not addressed in this study, it would be of interest to compare rates of invasive management in patients with and without cancer. Whether patients with cancer are less likely to receive invasive management due to a perceived higher risk of complications and if this may have contributed to the observed higher mortality warrants further study.

Thirdly, cancer patients represent a vastly heterogeneous population, and as such the synthesis of data in this field must be done carefully in order to maintain consistency. As cancer patients have been traditionally excluded from large-scale cardiovascular trials, high quality data is limited in this population. This further justifies the current publication as it provides a succinct summary of the available evidence, limited as it may be.

There are two major limitations to this study. First, the generalizability of some results is limited by the high degree of statistical heterogeneity between studies resulting from the clinical heterogeneity of the examined population and trial design. In particular, despite a statistically significant increase in all-cause mortality measured in-hospital and at one-year in cancer patients, there was high statistical variance between studies. Second, the lack of patient level data, including cancer type and the time interval between cancer diagnosis and ACS limits the interpretability of the findings. For example, the difference in survival is highly dependent on the cancer type, tumour stage, grade and biology [11]. Additionally, the treatment of the cancer itself may have indirect cardiac risk, such as increased bleeding propensity which has important prognostic implications [12].

## Conclusion

With the vast improvement in cancer prognosis over recent decades, focus must be given to the optimal management of cardiovascular disease in the survivorship population. Patient-specific assessment of risk, accounting for disease characteristics and treatment, and close communication with oncology providers is vital in defining optimal treatment strategies in this population.

## Data Availability

Not applicable.

## Abbreviations

ACS: Acute coronary syndrome
PCI: Percutaneous coronary intervention
STEMI: ST elevation myocardial infarction
